# Liquid electron microscopy: then, now and future

**DOI:** 10.1186/s42649-019-0011-7

**Published:** 2019-10-25

**Authors:** Anahita Vispi Bharda, Hyun Suk Jung

**Affiliations:** Division of Chemistry and Biochemistry, College of Natural Sciences, Kangwon National University, Chuncheon-si, Gangwon-do 24341 South Korea

**Keywords:** Liquid-phase, Electron microscopy, TEM, STEM, Nanoparticle

## Abstract

Contemporary microscopic imaging at near-atomic resolution of diverse embodiments in liquid environment has gained keen interest. In particular, Electron Microscopy (EM) can provide comprehensive framework on the structural and functional characterization of samples in liquid phase. In the past few decades, liquid based electron microscopic modalities have developed tremendously to provide insights into various backgrounds like biological, chemical, nanoparticle and material researches. It serves to be a promising analytical tool in deciphering unique insights from solvated systems. Here, the basics of liquid electron microscopy with few examples of its applications are summarized in brief. The technical developments made so far and its preference over other approaches is shortly presented. Finally, the experimental limitations and an outlook on the future technical advancement for liquid EM have been discussed.

## Introduction

Transmission electron microscopy (TEM) involving electron scattering based optical imaging has been practiced worldwide since the early twentieth Century. TEM offers powerful capabilities of imaging that can be reproduced to a broad range of disciplines like physical, chemical, material and life sciences. In biology, it is implicated with the ability to delineate critical structural, functional and compositional information of heterogeneous specimens at near-atomic resolution. In case of material sciences, TEM imaging established finer insights on the atomic rearrangements, defects, nano-structure dynamics and various structure-behavior characteristics. Whereas physical science has adopted TEM imaging to study energy transduction and probe based systems in a more comprehensive manner with better resolution. Inside the electron microscope, only those samples which are thin enough for easy electron beam absorption-transmission and able to withstand harsh vacuum conditions are resolved to achieve high resolution images.

TEM based nanoscale visualization in structural biology is particularly constrained to stationary, fixed/embedded, stained or frozen samples that involve additional methods of sectioning, slicing or even fractionation of cells (Peckys and de Jonge 2014; Robinson et al. 2007; Stahlberg and Walz 2008; Pierson et al. 2009; Hoenger and Bouchet-Marquis 2011; Kourkoutis et al. 2012; Ruska 1942). Currently, EM techniques are employed to undertake solid faceted mechanisms at near-atomic resolutions including Transmission Electron Microscopy (TEM), Cryo-Electron Microscopy (Cryo-EM), Scanning Transmission Electron Microscopy (STEM), 4D Electron Microscope as well as Correlated Light and Electron Microscopy (CLEM) (Meyer et al. 2008; Chung and Jung 2016; Kühlbrandt 2014; Buban et al. 2009; Zewail 2010; Sartori et al. 2007). Generally, the architecture of water or fluid containing different biological macromolecules like proteins, nucleic acids, membranes and cells is definitive. During sample preparation for EM, the conversion from liquid to solid state causes changes in the overall conformational and structural properties of these biological molecules resulting in the lack of resemblance from their realistic forms (Gilmore et al. 2013). Additionally, imaging of immobilized samples cannot be used to study dynamic processes and imposes significant issues in real-time data extraction. In bioscience, this leads to an imprecise interpretation of active mechanisms from their respective stationary structures. In order to preserve the samples from undergoing internal bond breakage or disordering in response to different buffer ingredients or radiation, cryo-electron microscopy has provided an outstanding mark in creating near-native specimens through plunge freezing (Dubochet et al. 1988). Notwithstanding, even strategies like vitrification which allow preservation of the macromolecules in their native state (Adrian et al. 1984), can only produce slow-time paced, snapshots that have to be combined to understand the entire trajectory of the biological event (Chen et al. 2013). Thus, in order to achieve real-time freestanding blueprint of the fundamental processes, the specimen needs to be examined under a non-static, non-frozen STEW, liquid state. In such scenario, liquid-phase electron microscopy serves to implement the imaging of biological entities in liquid form without the need for any specialized sample-state conversions.

### Evolution of electron microscopy in liquid

Electron based imaging of wet, hydrated specimens has been dated right from the commencement of electron microscopy practice (Marton 1934; Parsons 1974). Liquid electron microscopy is a rapidly flourishing approach utilizing the potential of electron microscopy to image and examine liquid or liquid containing samples. However, the incompatibility of maintaining a liquid environment under the vacuum condition hinders its thorough application. Under such system, it becomes difficult to keep the sample in their native state or wet form for true examination, since the internal gas has been replaced by vacuum in the microscopic column. Also, heterogeneity in the chemical composition of molecules that uphold weak interactions affect the overall contrast in the final image output. Consequently, even though liquid electron microscopy has notable advantages and roles in diverse branches of science, it experiences some drawbacks. Keeping that in mind, over the past decades liquid EM has seen manifold developments in the technicalities to achieve better spatial and temporal resolution besides better reproducibility in aqueous solution. Here, we describe an outlook on the revolution of liquid electron microscopy with significant improvements in the experimental design that can be used to provide indispensable information against diverse scientific problems.

Execution of successful imaging of samples in liquid state has been undertaken by various modalities of electron microscope. These include TEM, Scanning Electron Microscopy (SEM), scanning TEM (STEM) and other inter-combinatory models like light microscope with SEM (Zheng et al. 2009; Grogan and Bau 2010; de Jonge et al. 2009; Nishiyama et al. 2010; Rehn and Jones 2018). Each of them administered distinctive strategies to generate images and achieve better liquid compatibility. One of the early approaches of liquid TEM involved the use of an open chamber system operating with pumping apparatus to ensure satisfying hydrated and pressure conditions (Ruska 1942). Here, care was also taken to manage proper vacuum status for optimum microscopic activity, not allowing evaporation or loss of the sample. However, such scheme remained inefficient to carry out experiments engaging migration or exchange of liquids. Not only that, but the use of thicker liquid layers further fabricated problems in smooth image processing owing to the scattering effect of the electron beams in TEM (de Jonge and Ross 2011). With technical advancement, these liberated or otherwise called the open cell chambers have now been applied to SEM to image ultrastructures of various biological elements in wet environment (Parsons et al. 1974; Daulton et al. 2002; Sugi et al. 1997). Employment of such open environment cell chambers proved pragmatic for imaging liquids in SEM, where there was no need for thin samples. This so-called environmental scanning electron microscopy (ESEM) has emerged as a viable approach in contemporary decades to show promising results to visualize aqueous cell surfaces (de Jonge and Ross 2011; Kirk et al. 2009). Hither, cells are primarily shifted to an environment containing cooled pure water where, a number of microbes are able to endure the transfer process enabling fulfilling results from ESEM imaging. However, eukaryotic cells suffer from a shock due to osmotic imbalance and the resulting pressure created induces grave changes in their innate structure and morphology leading to cell rupture and death. It is thus important to arrive at a compromising condition so as to achieve both intact cellular integrity as well as microscopic examination. Hence, ESEM provides reasonable imaging of the pristine surfaces of various microbes and other non-eukaryotic systems in pure water. However, in case of eukaryotes only the ones that are fixed yet hydrated can be visualized effectively (Kirk et al. 2009).

Since the resolution achieved was accountable only to the surface linings or less bulky specimens, a second approach of closed-cell microscopy was constructed. Unlike the open environmental chamber, in liquid cell system electron-transparent membranes were used to seal the chamber containing a thin film of liquid sample (Parsons 1974; Abrams and McBain 1944). Generally, a liquid cell is composed by etching two microchips made up of semiconductors like silicon (Si) that possess an electron transparent window mostly that of amorphous silicon nitride (SiN). One of the first reports of probing the internal activity such as Cu deposition inside a closed-liquid electrochemical cell via TEM using SiN membranes paved the way for a new dimension in liquid microscopy (Williamson et al. 2003). With time, there have been outstanding developments in liquid cells with better microfabrication technology aiming for thinner membrane apparatus (de Jonge and Ross 2011). Microfabrication as a whole fixes the key issues existing in a closed liquid cell by providing high durability, reproducibility and easy handling of the specimens. STEM has gained much importance in terms of generating good contrast, better resolution images from high atomic number (Z) materials like nanoparticles (NPs) with heavy metals or thicker liquid films (de Jonge et al. 2009; Ring and de Jonge 2010; de Jonge et al. 2010; Peckys et al. 2009). Relatively, all microfabricated cells utilize thin beam-transparent SiN films as window material for support and resistance to pressure between the internal cell arena and the microscopic vacuum. Conjunctional light and SEM microscopy is capable of maintaining the cells under observation in a fully hydrated state (Nishiyama et al. 2010). One report showed the application of affinity biofilms as a coating on the surface of the liquid cells for 3D structural determination of simian rotavirus double-layered particles (Dukes et al. 2013). By appropriate usage of the microfluidic holder and affinity capture approach for association of the particles with the chamber, dynamic visualization could be realized with lesser degree of the diffusion of the particles in the liquid environment.

### Advancements in liquid EM technology and instrumentation

Dating from the past, in pursuit of bringing about developments in the TEM based liquid studies, thin aluminum foils were employed to perceive the ultrastructures of a wet biological sample (Marton 1934). However with limitations in this aspect, there was a need for a more elaborative approach to explore the liquid-dynamics at a profound level. The new era of liquid electron microscopy is brimming with significant breakthroughs made to combat the existing technological difficulties like resolution, signal-to-noise ratio (SNR), radiation damage and spatial-temporal resolution keeping into account the specimen characteristics, geometry along with microscopic efficiency (Bogner et al. 2007). Although there have been varied attempts in designing the cells, some criticisms still remain to be resolved. These include knowing about the different material type that can be used for tight sealing in a vacuum microscopic arena, the region where the liquid-phase reaction and interaction occur and finally the windows imparting transparency to electron beam with low contrast effects for fair visualization. One of the key attempts in this regard was the implementation of spherical and chromatic aberration corrections which has inherently revolutionized not only TEM based studies but also among a newer branch involving cryoimmersion light microscopy (de Jonge et al. 2019; Kim et al. 2018; Faoro et al. 2018). As a result of such contrast delocalization, the artifacts can now be averted resulting in rigorous apprehensions made towards nanoparticle studies. Efforts have been taken to formulate nanofluidic liquid-cell in TEM known as nanoaquarium in order to image such NP aggregations (Grogan and Bau 2010). By allowing in situ electron microscopy, such cells allow real-time observations of different liquid immersed nanostructures with high resolution. Also, advances in electron optics and detectors are applied to reduce the electron dose needed and to improve the resolution (Peckys and de Jonge 2014; Ross 2015).

With the development of microfluidic specimen holders, sealed environmental chambers can now be made with the help of microchips providing movement of liquid in the microscopic apparatus. This design was adopted in a study wherein a liquid flow holder system manufactured by Protochips Inc. was intended to deliver TEM imaging for gold nanoparticles in a thin layered saline water environment (Klein et al. 2011). Further, improvisation of these liquid cells led to the use of graphene as a thinner window membrane material (Cho et al. 2017). The advantage of using graphene cells was evident from a more desirable display of spatial resolution owing to minimal scattering of the bombarded electrons (Yuk et al. 2012). Furthermore, collaborative approaches amalgamating Liquid EM with other modalities like that of light or fluorescent microscopy has found to be efficient to provide a detailed insight of the organization of proteins and their structure at good resolution. One of such work has led to the understanding of the conformation, localization and distribution patterns of HER2 in the plasma membrane of SKBR3 breast cancer cells using ESEM with STEM at spatial resolution of 3 nm (Peckys et al. 2015).

### Advantageous potential of liquid EM

Since its inception, liquid electron microscopy has occupied an important place in molecular research, bestowing an exclusive platform for a broad range of scientific background. In the realm of imaging, it has created new perimeters in exploring the regions that were not capable had it been the conventional electron microscopy. Recently with improvised technological experimentations, liquid EM has penetrated in material-physical studies involving complex phenomena such as formation, growth, transformation, etching, nucleation and interactions of nanoparticles in solvated systems (Kim et al. 2018; Lee et al. 2017; Zhu et al. 2018). Moreover, the use of Graphene in closed-liquid cell has gained momentum owing to its powerful advantage as an optimum window material for atomic resolution TEM or STEM (Cho et al. 2017; Yuk et al. 2012; Textor and de Jonge 2018; Au-Chang et al. 2019; Yuk et al. 2014). This has led to a cascade of successive studies easing the way for an efficient liquid-handling protocol. Consequently, 3D structural features of platinum (Pt) nanocrystals were determined at atomic level using hybrid approaches combining graphene liquid cells, TEM and single particle based reconstruction analysis (Park et al. 2015). Another application proved the real-time visualization efficiency of liquid-phase TEM to monitor hollow Bismuth nanoparticles (Niu et al. 2013). Further, liquid STEM has been readily adopted to study gold NPs from the topmost zone of liquid layer (Schuh and de Jonge 2014).

A number of electrochemical experiments involved the use of open and liquid microscopy to entail more about the spatial and temporal information. This has also allowed to study anodes and cathodes using non-aqueous, nonvolatile ionic liquids that are relatively more compatible with the vacuum environment (Huang et al. 2010). It is now easier to highlight essential fundamentals like electro-deposition, battery reactions and corrosion processes to delineate the minutest of information using liquid phase electron microscopy as well as STEM (Jiang et al. 2014; Holtz et al. 2014; Ahmad et al. 2018). The ability to image liquid biological entities with a view to understand its underlying structural and functional aspects have led to giant strides in scientific discoveries. Liquid electron microscopy is positioned well to examine various cells and cellular structures at good resolution through thick liquid arrangement. It is now able to identify tagged structures, tracking cellular or macromolecular kinetics and even provide insights of unfixed-free floating cells in a conditioned liquid compartment. For example, one study demonstrated STEM derived imaging of the uptake of nanoparticles by eukaryotic cells in a defined microfluidic device. Furthermore, it exploited gold-NPs labelled epidermal growth factor (EGF) particles docking the EGF receptors from fixed COS7 fibroblasts grown on semiconductor based microchips in similarly enclosed chamber (de Jonge et al. 2009; Ring et al. 2011; Arkhipov et al. 2013). Correlative light and electron microscopy combining the signals from electron microscopy and fluorescently labelled probes can produce good spatial and temporal resolution and establish meaningful structural to functional analysis (Nishiyama et al. 2010). Such diverse studies impart profound knowledge at molecular level among the fixed or live cells. Determination of stoichiometric profile of proteins, their macromolecular conformers can be correlated well to functional characterization with a quicker reproducibility of data from enormously present cells (Peckys et al. 2013). Integrated methods using liquid TEM and energy-dispersive X-ray spectroscopy (EDS) have been successful in discerning chemical composition of nanoparticles as well as that of alloys (Lewis et al. 2014; Zaluzec et al. 2014). Comparing the two TEM imaging techniques i.e. in situ and ex situ can help display the level of competence provided by the former methodologies currently being studied in nanoscaled structure based chemical activities. Thus liquid EM, either in amalgamation or with modified instrumentation has been compelling to produce relevant interpretation about the objects under scrutiny with prudent resolution.

Interestingly, there are reports that show direct imaging of the native protein structures at ambient temperatures rather than 98 K in ice using TEM. With the rational feasibility of an appropriate aqueous condition and the advantage of an independent protein molecule free of labeling, it may be possible to unravel dynamics in protein biology. This facility escalates the capability of TEM to image unstained or unlabeled targets in water and yet achieve near-atomic resolution (Mirsaidov et al. 2012).

### Future prospects

As liquid electron microscopy assimilates critical improvements in performance and instrumentation, it encourages a better analysis to resolve many unanswered questions across various sciences. The development of liquid cell technology with different types of microfabrication options has opened a new frontier in attaining real-time visualization of micro and nano sized structures. It is implicated with deriving unique information that once seemed obscure.

There are paradoxical views on whether live imaging using fluorescently-labeled tags actually represent the cells being alive upon radiation exposure (de Jonge and Peckys 2016). The perplexity of reproducing realistic data still remains questionable. It is therefore important to arrive at simulations that are providing strong evidences about the dynamic processes that are under observation. The focus should be given to lower the magnitude of radiation doses at several folds. Hence more experimental friendly approach is the need of the hour. Likewise, a better understanding of the interactions between the sample and the beam in liquid environment can be produced to assess the best way to preserve the sample integrity. Another factor that needs to be taken into consideration is the avoidance of using tagging protocols that could also be involved in causing some degree of amendments in the concerned molecule (Mirsaidov et al. 2012). An environment undisturbed by such refitting will be more preferable for pragmatic conclusions.

Liquid electron microscopy with its other counterparts, with realized achievements has shown great potential in conveying pertinent intelligence of microscopy in a relatively native niche and among a wide range of samples. It is anticipated to flourish in the near future with unexpected discoveries keeping into account of the amount of importance it has received in the past few decades.

## Conclusions

Since the evolution of electron microscopy, ardent endeavors have materialized to execute easy imaging of water and other solutions (Marton 1934). Gradually, liquid phase electron microscopy has thrived with key findings mitigating the field of microscopy. While conventional transmission electron microscopy intends to provide imaging of thin fixed or vitrified specimens, liquid phase electron microscopy casts an entirely different approach to understand molecules under a liquefied system. In modern times, the use of new technical adjunctions like that of microfabrication have reformed the possibility of deriving liquid phase image acquisition supplementing legitimate information (Cho et al. 2017; Radisic et al. 2006). It is expected to flourish in near future with exciting new developments coming in its way. With ever expanding application of in a broad range of fields, it is anticipated that liquid electron microscopy will pose to be a great analytical tool solving a number of molecular and biomolecular related questions.

## Data Availability

Not applicable.

## Abbreviations

3D: 3-Dimensional
4D: 4-Dimensional
CLEM: Correlative Light and Electron Microscope
cryo-EM: cryo-Electron Microscopy
EDS: Energy-dispersive spectroscopy
EGF: Epidermal growth factor
ESEM: Environmental Scanning Electron Microscopy
K: kelvin
NP: Nanoparticle
SEM: Scanning Electron Microscopy
SiN: Silicon Nitride
SNR: Signal-to-noise ratio
STEM: Scanning Transmission Electron Microscopy
TEM: Transmission Electron Microscopy
